# Antibiotic Resistance Genes in Agricultural Soils: A Comprehensive Review of the Hidden Crisis and Exploring Control Strategies

**DOI:** 10.3390/toxics13040239

**Published:** 2025-03-24

**Authors:** Yuanye Zeng, Runqiu Feng, Chengcheng Huang, Jie Liu, Fengxia Yang

**Affiliations:** 1 Agro-Environmental Protection Institute, Ministry of Agriculture and Rural Affairs, Tianjin 300191, China; zengyuanye@foxmail.com (Y.Z.); 18190690768@163.com (C.H.); 2 College of Pastoral Agriculture Science and Technology, Lanzhou University, Lanzhou 730020, China; fengrq20@lzu.edu.cn (R.F.); jieliu@lzu.edu.cn (J.L.); 3 Agro-Ecosystem, National Observation and Research Station, Dali 671000, China

**Keywords:** agricultural soil, antibiotic resistance genes, sources, potential risks, control strategies

## Abstract

This paper aims to review the sources, occurrence patterns, and potential risks of antibiotic resistance genes (ARGs) in agricultural soils and discuss strategies for their reduction. The pervasive utilization of antibiotics has led to the accumulation of ARGs in the soil. ARGs can be transferred among microorganisms via horizontal gene transfer, thereby increasing the likelihood of resistance dissemination and heightening the threat to public health. In this study, we propose that physical, chemical, and bioremediation approaches, namely electrokinetic remediation, advanced oxidation, and biochar application, can effectively decrease the abundance of ARGs in the soil. This study also highlights the significance of various control measures, such as establishing a strict regulatory mechanism for veterinary drugs, setting standards for the control of ARGs in organic fertilizers, and conducting technical guidance and on-farm soil monitoring to reduce the environmental spread of ARGs and protect public health.

## 1. Introduction

Antibiotics, which are natural secondary metabolites generated by bacteria and fungi, function as either growth inhibitors (bacteriostatic) or substances that can kill bacteria or fungi (bactericidal) [1]. Antibiotics are of great significance in preventing and treating animal diseases, enhancing animal performance [2], and comprehensively controlling crop diseases [3,4]. Consequently, they are extensively used in animal husbandry and agriculture. However, the emergence of antibiotic resistance genes (ARGs) has been brought about using antibiotics. During the evolution of microorganisms, ordinary genes encoding proteins are activated and transformed into resistance genes that are adapted to the presence of antibiotics or are capable of metabolizing antibiotics [5]. Excessive antibiotic use has exerted selective pressure on microbial communities, driving the proliferation of ARGs and making infections difficult to treat. Furthermore, ARGs have been detected in various environmental sources such as wastewater, agricultural soil, animal feces, and hospital waste [6,7,8,9,10].

ARGs disseminate through multiple pathways. Horizontal gene transfer (HGT) primarily drives transmission between bacteria through mobile genetic elements (plasmids, integrons, and transposons) in a replication-independent process [11]. Other mechanisms include vertical transmission (inheritance during bacterial division), transformation (environmental DNA uptake), and transduction (phage-mediated transfer) [12]. Selective pressures from antibiotic overuse and pollution accelerate ARG proliferation by favoring resistant bacterial populations [13,14]. Additionally, host adaptation influences ARGs stability and expression: the physiological state of host bacteria and environmental adaptations determine how efficiently resistance genes are maintained and activated [15]. A critical consequence of ARG dissemination is multidrug resistance (MDR), where bacteria develop resistance to multiple antibiotics simultaneously [16]. MDR complicates infection treatment and exacerbates the challenge of controlling ARG contamination [17].

Given the accelerated development of China’s livestock and poultry farming industry, its scale is constantly expanding. Consequently, there is an ongoing need to enhance the industry’s quality, efficiency, and competitiveness. Antibiotics are used for animal treatment in this industry [18]. Since antibiotics have entered the livestock industry, there has been a rapid increase in their use of veterinary antibiotics in livestock farming. In livestock farming in particular, over two-thirds of the global antibiotic supply is used in agricultural production, as studies have shown [19]. Antibiotic overuse has prompted awareness of environmental pollution and harm, with a 55.4% decrease in antibiotic usage in China from 2014 to 2019 [20]. Livestock manure is an important organic fertilizer, and studies have indicated that its prolonged application enhances soil bacterial diversity, thereby mitigating the deleterious effects of chemical fertilizers on soil quality [21]. The utilization of organic fertilizers plays a pivotal role in enhancing soil fertility, promoting soil biodiversity, and augmenting crop yield and quality [22]. However, the use of antibiotics in agriculture continues to contribute to antibiotic pollution despite a gradual decline in their application [23]. Livestock manure fertilizer is a significant source of antibiotics in agricultural soils [23]. It has been demonstrated that persistent selection pressure resulting from the increased misuse of antibiotics fosters the prevalence of acquired resistance to a range of clinically relevant pathogens and commensal bacteria in the soil, thereby transforming the soil environment into a significant reservoir of ARGs [24,25]. Xu et al. (2021) found that the dissipation of ARGs in soil is more pronounced under anaerobic conditions than under aerobic conditions [26]. Another study analyzed 285 ARGs in soil samples from 1012 sites worldwide, creating the first global map of ARG distribution in surface soils [27]. These studies suggest that ARGs may enter soil ecosystems through livestock manure, and that soil ecosystems are becoming hotspots for the dissemination of ARGs.

## 2. Source, Occurrence and Hazards of ARGs in Agricultural Soils

### 2.1. The Sources of ARG Contamination in Agricultural Soils

**Figure 1:**
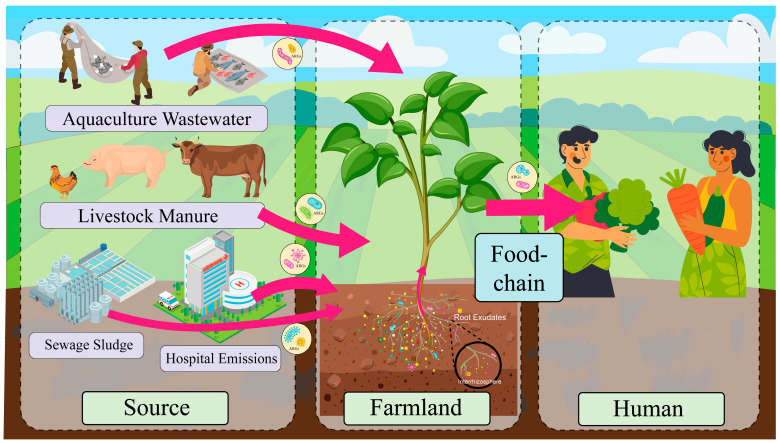
Sources and spread routes of antibiotic resistance genes (ARGs) in agricultural soils. Livestock and poultry manure, aquaculture wastewater, sewage sludge, hospital discharge, etc., are the main sources of pollution. ARGs from sewage sludge may enter the soil through its application as organic fertilizer, while hospital emissions (e.g., wastewater and airborne particles) can reach agricultural environments via irrigation with contaminated water or atmospheric deposition. These pathways facilitate the transmission of ARGs in the agricultural environment, posing potential threats to soil ecology and human health. (Some of the images in this figure were designed by Freepik).

There are multiple factors contributing to ARG contamination in agricultural soils. These encompass the use of animal manure, the release of ARGs from aquaculture facilities, hospitals, and pharmaceutical firms, and the application of agricultural-origin sludge [28]. Antibiotic overuse in healthcare and agriculture creates selective pressure that drives the evolution of resistant bacteria, undermining the efficacy of antimicrobial therapies [29]. Animal manure and sewage sludge in agricultural practices are employed to enhance soil fertility. However, these practices facilitate the dissemination of antibiotic-resistant bacteria (ARB) and genes from animal manure to farmland soil. When cow manure, chicken manure, or pig manure, which may contain ARB and ARGs, is applied to agricultural soils to enhance fertility, it directly introduces these contaminants. Several studies have identified cow manure, chicken manure, pig manure, and sewage sludge as the primary sources of antibiotics and ARG contamination in agricultural soils [30]. In addition to the utilization of livestock manure from farms as organic fertilizers, the use of antibiotics in aquaculture can result in the entry of these compounds into agricultural soils through water discharge [31]. The use of antibiotics in human medicine has also been identified as a contributing factor to the selection of ARGs in bacteria, which can spread through the environment via treated municipal wastewater and biosolids. These biosolids can contaminate agricultural land when discharged [32]. Sludge agro-use, defined as the application of sludge from municipal wastewater treatment to agricultural land with the aim of enhancing soil fertility and improving soil structure, is of particular concern. However, this practice may result in the contamination of ARGs in agricultural soils due to the presence of antibiotic residues and antibiotic-resistant genes in sludge [33]. The sources and spread of ARGs in farmland soils are shown in Figure 1. Additionally, the transmission of antibiotic resistance genes via airborne pathways has been documented. It has been demonstrated that environmental bacteria can be present in the atmosphere in the form of bioaerosols, which are adsorbed on the surface of particles [34]. These bioaerosols can contain ARGs, which can return to the land surface through precipitation and snowfall, contaminating the Earth’s entire surface [35]. Therefore, air pollution may be one of the sources of ARGs. Different agricultural practices and changes in the surrounding environment have the potential to introduce antibiotic resistance genes into agricultural soils, thereby posing a risk to the agricultural environment and public health.

### 2.2. Occurrence Patterns of ARGs in Agricultural Soils

ARG contamination in agricultural soils exhibits significant diversity. A study by Zheng et al. (2022) reported the detection of 558 ARGs in 1088 soil macrogenomic samples worldwide, with a higher prevalence of ARGs observed in agricultural habitats compared to non-agricultural habitats [36]. In a related study, Zeng et al. (2019) concluded that the relative abundance of nine ARGs in greenhouse and open field soils spanned five orders of magnitude, ranging from 1.15 × 10^−7^ to 9.78 × 10^−2^ copies/16S rRNA copies [37]. It is therefore evident that due to the high abundance of antibiotic resistance genes in agricultural soils, there is diversity in the contamination they introduce into agricultural soils. Studies have identified sulfonamides, tetracyclines, fluoroquinolones, macrolides, and their corresponding ARGs as the predominant types of contamination [28,30]. The relative abundance of multidrug, sulfonamide, and tetracycline resistance genes ranged from 10^−4^ to 10^−3^ copies/16S rRNA gene copies, with *sul*1 and *sul*2 genes being the most prevalent sulfonamide resistance genes and *tet*M, *tet*W, *tet*O, and *tet*X being the most abundant tetracycline resistance genes [30]. According to a study by Fang et al. (2023), the median antibiotic concentrations in agricultural soils spanned from 0.008 to 160.0 μg·kg^−1^ [28]. However, in comparison with the median values in sewage sludge (100–1000 μg·kg^−1^) and in livestock manure (10–100,000 μg·kg^−1^), those in agricultural soils were markedly lower [38,39]. Despite the low antibiotic residue concentrations in soil environments, these residues can persist in soil for extended periods, often coexisting with multiple antibiotic classes. This phenomenon can generate selection pressure, leading to the induction of multiple resistance genes. Ultimately, this can result in a broad array of antibiotic resistance genes contaminating diverse types of environmental samples, as evidenced by recent studies [40]. Contamination levels of ARGs in farmland soils vary geographically. Thirteen ARGs were detected in agricultural soils from Enshi, Hubei, a region with high selenium levels (geometric mean of agricultural soils was 2.41 mg·kg^−1^), and the multidrug resistance genes *ade*F, *mtr*A, and *poxt*A, the aminoglycoside resistance gene *rps*L, and the sulfonamide resistance gene *sul*2 were enriched in selenium-enriched systems [41]. In a related study, Qiao et al. (2021) investigated farmland situated in the vicinity of gold tailings, located 57 km northwest of Beijing, China. Their analysis led to the detection of 75 ARGs in soil samples, encompassing 327 distinct isoforms [42]. Heavy metals can induce cross-resistance mechanisms (e.g., efflux pumps) or co-select for resistance genes through shared genetic platforms (e.g., integrons, plasmids) [43]. This phenomenon is particularly relevant in agricultural soils amended with metal-rich manure or irrigated with contaminated water [44]. Cheng et al. (2021) found that a total of 149 ARGs and 28 MGEs were detected in farmland soils near the phosphorus base of Shifang City in Sichuan Province [45]. Yang et al. (2023) found that the levels of *qnr*A, *qnr*D, *aac*(6′)–Ib–cr, *sul*2 and *dfr*A were higher in tidal flat reclaimed farmland soils used for land demand along the Yellow Sea coast than in other land uses [46]. A study by Li et al. (2020) focused on examining the presence of ARGs in soil samples sourced from black soil farmland in northeastern China. Their findings revealed the presence of a total of 178 ARGs, including mobile genetic elements (MGEs). Furthermore, the diversity and abundance of these ARGs significantly increased in response to manure application [47]. Song et al. (2023) examined agricultural soils in the Tibetan Plateau and detected ARGs in quantities ranging from 5.66 × 10^5^ to 6.22 × 10^7^ copies per gram of soil, which is higher than those seen in previous Tibetan land use [48]. These findings highlight significant regional variations in ARG contamination levels, which are influenced by climatic conditions and environmental factors such as heavy metal pollution.

### 2.3. Potential Hazards of ARGs Contamination in Agricultural Soils

The presence of antibiotics in the agricultural soil environment has the potential to pose environmental hazards and health risks. When antibiotics are taken up by plants, they can be transported, as can the ARGs, through the plant’s vascular system (xylem and phloem) to aboveground tissues such as branches, leaves, and fruits [49]. Concurrently, plant rhizosphere microorganisms interact with ARB in soil, potentially leading to the colonization of plants by endophytic bacteria carrying ARGs [50]. Through the food chain, humans and other animals can be infected with these bacteria when plants consume them. It has been demonstrated that *rmtB*-positive bacteria in agricultural soils can be transmitted in swine farms and their environments, and that these ARB or ARGs can be acquired by humans through the food chain [51]. Zhang et al. (2024) established a soil–lettuce–snail ecological chain. They used genetically engineered fluorescent *Pseudomonas aeruginosa* with the multidrug-resistant plasmid RP4 to trace it through the food chain. Findings showed that exogenous *Pseudomonas monocytogenes* could move from the soil into the plant interior and then into the snail gut. Moreover, horizontal gene transfer from the RP4 plasmid in the bacteria was detected [52]. This finding indicates that antibiotic resistance genes present in agricultural soils have the potential to be transmitted through the food chain. Furthermore, the presence of these genes has been demonstrated to disrupt the natural ecological balance of microbial communities, which can lead to adverse effects on soil health and function.

Livestock manure application alters soil aggregate structure and microbial communities [53], while simultaneously introducing bacterial pathogens, VFGs, and ARGs into soils [54]. Antibiotic dissipation enhances soil respiration, ammonification, and nitrification activities [55], yet their residual maintains selective pressure for resistance evolution. Mobile ARGs in animal manure (e.g., chicken) transfer between bacteria, conferring resistance to pathogens—particularly plant-pathogenic strains—and enhancing their ecological competitiveness. Notably, these resistant strains (which are predominantly multidrug-resistant) persist in ecosystems, with even single-class resistance exacerbating contamination risks [56]. ARGs absorbed by crops can spread through the food chain, threatening human health and ecological stability, thereby underscoring the urgency of controlling agricultural ARG spread.

## 3. Dissemination and Influencing Factors of ARGs in Agricultural Soils

### 3.1. Variation in ARGs Under Different Soil Types

A recent increase in the number of studies on ARGs in soil has been observed. These studies have revealed that there are differences in the contamination and fate of ARGs in different types of soils, thereby underscoring the complexity and diversity of ARG contamination in diverse soil environments. For instance, Macedo et al. (2020) demonstrated that the persistence of ARGs varied across sand, clay, and peat, with the type of genes and the viability of the bacteria carrying these ARGs in the soil being pivotal factors [57]. A study by Seyoum et al. (2021) found that when treated wastewater was used for the irrigation of agricultural fields, low-clay soils (loamy soils) usually had higher concentrations of antibiotic residues than high-clay soils (loam and clay), and tetracycline (TC) and chlortetracycline (CTC) levels were significantly higher in wastewater-irrigated loamy sand [58].

The time required for ARGs to decay to background levels in bare loam soils following the application of swine wastewater was shorter in sandy loam and longer in silt loam. However, soil type did not appear to affect the decay of soil ARGs after the application of swine wastewater [59]. Eric et al. (2019) measured the levels of 10 ARGs in silt loam, chalky clay, and chalky clay loam soils and found that, with the exception of silt loam *erm*B and *tet*M, the amount of ARGs increased after manure application before sowing, while the other variables were not affected [60]. Taken together, these studies demonstrate that the differences in antibiotic resistance gene contamination in different types of soils depend on the type of contamination and the genotypes of the genes, and have less of a correlation with whether the soil itself is clay, sand, or loam.

The physicochemical properties of soils are determined by their type, with different soil types exhibiting different pH levels ranging from acidic to alkaline, and varying levels of fertility. Soil properties influence the structure and function of microbial communities through various mechanisms, which in turn affect the distribution and spread of ARGs. A study demonstrated that the acidity and alkalinity index (pH), the salinity index (EC), and the nutrient index (TN) in the soil environment significantly influenced the variation and distribution of ARGs [61]. The study did not delve into the specific mechanisms by which pH affects ARGs; however, it is well established that changes in soil pH have a general impact on the composition and activity of microbial communities, as well as the relative abundance and diversity of bacteria [62]. Consequently, these changes may also influence the distribution and abundance of ARGs. Physicochemical factors such as organic matter, nitrogen content, and heavy metal content in soil have been demonstrated to have significant effects on ARGs. Specifically, organic matter has been shown to provide nutrients to microorganisms and increase microbial biomass, which in turn may increase the abundance of ARGs. Concurrently, the presence of heavy metals has been observed to induce microorganisms to transfer antibiotic resistance genes, thereby increasing the abundance of resistance genes in response to heavy metal stress [63]. The metabolic pathways of microorganisms and the expression of ARGs can be influenced by the redox status of soil, for which the redox potential (Eh) functions as an indicator [64]. This dynamic relationship is characterized by the adoption of distinct metabolic strategies by microorganisms in the face of varying redox conditions [65]. The outcome of this interaction, namely the coexistence or exclusion of microbial populations, is determined by the metabolic strategies employed by these microorganisms [66]. The symbiotic patterns of soil microorganisms are altered, and the bacterial connectivity among them is affected, which in turn affects ARG and MGE abundance [67,68]. The dissemination of ARGs among microbial communities is facilitated by various mechanisms, including horizontal gene transfer [69]. Additionally, the composition of soil microbial communities and their metabolites can modulate soil chemistry [70], thereby indirectly influencing the behavior and fate of ARGs.

### 3.2. Influence of Cropping Systems on the Distribution and Spread of Soil ARGs

As a pivotal element of soil ecosystems, distinctions in cropping systems have the capacity to instigate alterations in soil physical, chemical, and biological characteristics, which, in turn, exert an influence on the structure and function of microbial communities [71]. These microorganisms are of paramount importance in soil ecosystems, participating in processes such as nutrient cycling, organic matter decomposition, and pollutant degradation [72,73,74]. As such, the effects of different tillage patterns on soil structure, microbial communities, and material cycling may further alter the transmission pathways, rates, and distribution of ARGs [75]. Importantly, tillage practices also significantly influence greenhouse gas (GHG) emissions (e.g., CO_2_ and N_2_O), with reduced tillage systems often promoted for enhancing soil carbon sequestration and mitigating climate impacts [76,77]. However, debates persist regarding the actual GHG reduction potential, as some studies report comparable emission levels between tilled and non-tilled soils under specific management practices [78,79,80]. For instance, FAO highlights the role of conservation tillage in reducing carbon loss [81], while others note context-dependent trade-offs in N_2_O fluxes due to altered nitrogen dynamics [82]. Furthermore, tillage patterns have important effects on soil compactness, moisture status, and temperature variability [83,84]. For instance, deep tilling and soil loosening enhance soil porosity, thereby facilitating soil aeration and the growth and colonization of aerobic microorganisms [85]. This phenomenon can potentially hinder the abatement of ARGs within these microorganisms and likely promote their spread. Additionally, deep tilling and soil loosening can augment the relative stability of soil temperature [86], thus creating more conducive conditions for plant root growth. The maintenance of a relatively stable temperature environment is conducive to the sustenance of microbial activity, which, to a certain extent, may influence the survival and dispersal capability of ARGs [87].The dynamics of soil moisture have been demonstrated to directly impact the metabolic activities of microorganisms and the expression of ARGs, with moist environments typically fostering microbial reproduction and the dissemination of resistance genes [88,89], while dry conditions may impede their activity.

Soil ARGs have been observed to be closely related to human activities, particularly land tillage, the selection of cropping patterns, fertilization, and irrigation [90]. As previously mentioned, soil tillage exerts a dual regulatory effect on the soil structure [91] and the composition of soil microbial communities [92]. This suggests a potential impact of soil tillage patterns on the living environment of antibiotic-resistant bacteria in the soil, which in turn affects ARGs in the soil. Long-term no-tillage land has been shown to significantly reduce the relative abundance and accumulation of a number of ARGs under dryland conditions compared with plowed soils [93]. Furthermore, agricultural soils serve as primary repositories for ARGs due to their exposure to multiple waste streams, including livestock manure, sewage sludge, and aquaculture wastewater [28,30,33]. While tillage activities may enhance ARG accumulation by disturbing soil microbial communities and increasing gene transfer opportunities [94], the elevated abundance and diversity of ARGs in agricultural soils are primarily attributed to continuous inputs of antibiotic-contaminated organic amendments. The continuous cropping pattern of monoculture crops has been shown to result in soil nutrient imbalance, reduced soil enzyme activities, reduced total soil microbial population, community structure and diversity, and the accumulation of transmitted pathogens [95,96]. In contrast, crop rotation systems, wherein diverse crops are cultivated at distinct intervals, have been demonstrated to play a pivotal role in regulating the antibiotic resistance profile of soil [97]. This phenomenon may be attributed to the influence of crop rotation on the variability of ARGs by modifying the structure and function of the inter-root microbial community [98]. Overall, tillage practices influence ARG abundance through indirect mechanisms, such as altering soil microbial biomass carbon, community structure, and HGT efficiency [99]. For example, deep tillage increases soil aeration and microbial activity, potentially promoting HGT via MGEs like plasmids [93]. Conversely, reduced tillage fosters anaerobic conditions that inhibit ARG proliferation by limiting bacterial conjugation [26,93]. These effects highlight the complex interplay between tillage, microbial dynamics, and ARG dissemination in agroecosystems.

### 3.3. Impact of Plant Root Activities on ARGs in Soil

The activities of farm crop roots play a pivotal role in the soil ecosystem, and their impact on soil ARGs has garnered significant attention in recent years. Research has demonstrated that there are some differences in the uptake of antibiotic resistance genes in soil by different crops. For example, wheat rhizosphere has been found to contain a richer array of ARGs, exhibiting both the highest abundance and diversity of resistance genomes [100]. In contrast, other crops such as barley, indica, and japonica also demonstrate some enrichment of resistance genomes, though not to the same extent as wheat. For instance, the relative abundance of β-lactam resistance genes in wheat inter-roots was found to exceed that of mycopeptide resistance genes, while phosphinomycin resistance genes were among the top three in indica rice rhizosphere [100]. The distribution characteristics of resistance genes in maize rhizosphere soils have been the subject of study. It was found that the abundance and diversity of ARGs varied in maize inter-root soils from different regions. *rbp*A, *vanR*O, and *mtr*A were mainly carried by native actinomycetes, and few mobile genetic elements were found in their flanking regions, suggesting that their mobility was low, whereas a portion of the *dfr*B genes, adjacent to recombination-binding sites (*attCs*), constituted mobile gene cassettes, facilitating the transfer of genes from soil bacteria to human pathogens [101]. Research in this area is limited, particularly in the context of legume crops. Chickpea (*Cicer arietinum*) forms symbiotic relationships with nitrogen-fixing rhizobia, which contribute to soil nitrogen availability and microbial community dynamics [102]. This process may indirectly influence ARG dynamics through two key mechanisms: (1) nitrogen fixation alters soil nutrient profiles, potentially favoring bacterial populations carrying nitrogen-metabolism-related ARGs [103,104]; (2) rhizobia themselves can act as reservoirs or vectors for ARGs, as demonstrated by studies detecting resistance genes in *Bradyrhizobium* strains [105]. However, the specific pathways by which legume symbiosis affects ARG abundance and transfer in soil remain poorly understood, warranting further investigation.

It has been demonstrated that the inter-root region of plants is a notable site for horizontal transfer of ARGs. The abundance of plasmids has been observed to exhibit a strong correlation with ARGs, suggesting that selective pressures exerted by plants on specific ARGs in the inter-root may result in the translocation of ARGs into conductor plasmids and their subsequent rapid dissemination among bacteria in the environment [100]. Therefore, crop root exudates are important factors that influence soil microbial communities and the distribution of resistance genes. The organic compounds present in root exudates can function as a nutrient source for microorganisms, thereby influencing their growth and metabolic activities [106]. This, in turn, affects the expression and dissemination of antibiotic resistance genes. For instance, the affinity of tetracycline for high-molecular-weight (≥10 kDa) rhizosphere exudates of aquatic plants contributes to the deposition of TCH in the roots, which enhances the expression of certain tetracycline-resistant genes (e.g., *tet*A) and the growth of tetracycline-resistant bacteria [107]. Ginger plants have been observed to exhibit resistance to the direct pressure of soil Cr and SMX by secreting humus-like exudates from their root system and interacting with inter-root bacteria. This interaction influences the uptake of heavy metals by crops and the selection of ARGs [108]. Plant root exudates significantly mitigate antibiotic resistance risks by reshaping rhizosphere microbial communities, suppressing potential hosts of ARGs, and reducing the abundance of MGEs that facilitate ARG transfer [109,110].

Furthermore, root exudates exhibit variability in composition and quantity across different crop species. Some crops may utilize root exudates to recruit microorganisms sensitive to specific antibiotics, while others may promote the proliferation of resistant strains. The expression and dissemination of ARGs may be promoted or inhibited by these exudates, contingent on the chemical composition of the specific compounds present. This dynamic interaction can potentially exert an impact on the composition and functionality of the soil microbial community. Consequently, the distribution and abundance of ARGs are affected. A study on root exudates from three distinct crops (lettuce, mustard, and maize) found that at the community level, lettuce inter-root bacteria exhibited enhanced growth in non-host root exudates, whereas mustard and maize inter-root bacteria demonstrated comparable growth in both host and non-host root exudates [111]. Bacterial growth activity varies across different crop types. While plant species richness enhances the metabolic activity of soil microorganisms, legumes negatively impact this activity, leading to divergent effects on ARGs [112]. While current research has focused on the response of individual strains to different root exudates, the actual inter-root environment is much more complex, with more complex microbial interactions. Therefore, the effects on ARGs need to be discussed in a categorized manner for in-depth research.

## 4. Reduction Strategies for ARGs in Agricultural Soils

As mentioned in Section 2.3 above, the presence of antibiotics and ARGs in agricultural soil poses environmental hazards and health risks: they can be absorbed by plants, be transmitted through the food chain, disrupt the natural ecological balance of microbial communities, and increase the risk of new resistant strains, with “silent reservoirs” contributing to multidrug-resistant pathogens emergence. All these indicate that the existence of antibiotic resistance genes in agricultural soil generates health and ecological risks via crop absorption and food chain transmission. The ARGs in agricultural soils pose a potential threat to the ecological environment and human health. It is therefore imperative to adopt effective reduction strategies. The development and implementation of a series of integrated reduction strategies to reduce the abundance and activity of ARGs in soil is an important measure for current environmental management and sustainable agricultural development. Several feasible reduction strategies are described below, including physical remediation, chemical remediation, bioremediation, and research on combined remediation techniques.

### 4.1. Physical Remediation Methods

Physical remediation techniques refer to the reduction in the spread and activity of ARGs through physical processes. Some studies have shown that anaerobic conditions can inhibit the interaction between ARGs and MGEs in the soil and reduce the potential risk of ARGs in farmland environments [26]. The adoption of reduced tillage practices, or the complete cessation of tilling, has been demonstrated to foster the development of anaerobic conditions within agricultural soils. This, in turn, has been shown to promote the enhancement of soil microbial diversity and metabolic activity while concurrently impeding the proliferation of ARGs within these environments [93]. Electrokinetic remediation of soil represents a distinct environmental remediation approach that utilizes an electric field to facilitate the extraction of pollutants from soil [113]. Li et al. (2018) found that the use of electro-remediation technology in contaminated soil effectively inactivated ARB in the soil and reduced TC-related ARGs [114]. Some studies have shown that ARGs can be effectively abated under high-temperature conditions by reducing genes encoding antibiotic efflux pumps, inactivating multidrug-resistant bacteria, and other mechanisms [115]. To this end, it is recommended that efforts be made to eradicate antibiotic-resistant genes in agroecosystems by treating soil at elevated temperatures.

However, physical remediation methods still have certain limitations. The creation of anaerobic conditions usually requires specific equipment and a longer period. For large-scale farmland soil remediation, implementation is difficult and costly. Electrokinetic remediation technology has certain requirements for soil texture and pollutant characteristics, such as soil conductivity. For soils with low conductivity or complex pollutant systems, the remediation effect may not be satisfactory. Although high-temperature treatment of soil can effectively reduce ARGs, it may also destroy the beneficial microbial communities and soil structure, affecting soil fertility and the subsequent recovery of ecological functions.

### 4.2. Chemical Remediation Methods

Soil chemical remediation is defined as the application of chemical methodologies to transform stable contaminants present within contaminated soils into less toxic forms that are non-hazardous to water bodies, plants, and humans [116]. Advanced oxidation techniques have emerged as effective methods for inactivating and eliminating ARBs and ARGs [117]. Advanced oxidation processes that rely on sulfate radicals have demonstrated efficacy in eliminating ARBs/ARGs through diverse activation methods [118]. Furthermore, there are currently techniques combining UV irradiation and chemical oxidation applied to wastewater treatment that have the potential for ARB inactivation and ARG degradation [119]. This comprehensive review underscores the potential for the future implementation of advanced oxidative abatement techniques in agricultural soils. Natural clay minerals, which possess the capacity to adsorb antibiotics and ARGs due to their substantial specific surface area, layered structure, surface charge, and cation exchange capacity, can be utilized as clay-based adsorbents to abate antibiotic-resistant genes in soil [120]. The employment of nanomaterials holds promise for enhancing environmental remediation, a prospect with the potential to yield substantial ecological benefits [121]. It has been demonstrated that zero-valent iron treatment can facilitate the removal of intracellular and extracellular ARGs by reducing potential host bacteria and *intI*1 [122]. The addition of soil amendments has also been demonstrated to contribute to the abatement of antibiotic resistance genes. Biochar, a prevalent soil amendment in agricultural settings, has demonstrated efficacy in this regard [123]. A study by Su et al. (2023) revealed that both block biochar and nano-biochar amendments were effective in controlling the proliferation of ARGs in soil, apart from *sul*1 [124]. Apart from biochar, other soil amendments have been shown to mitigate ARGs in the soil. For illustration, guano stone-loaded zeolite amendment has been demonstrated to effectively mitigate the mixed contamination of heavy metals, antibiotics, and ARGs in microplastic-contaminated soil [125]. Judicious selection of appropriate soil amendments can effectively counteract the presence of these contaminants, thereby safeguarding the integrity and quality of the soil matrix.

Despite the unique advantages of chemical remediation methods in dealing with ARGs, they also inevitably have some limitations. Advanced oxidation technologies may produce some by-products that could cause secondary pollution to the soil environment and ecosystems. Clay mineral adsorbents have limited adsorption capacities. For soils with high concentrations of ARGs, large amounts of clay minerals may be needed, and the disposal of the adsorbents after use is also a problem. Although nanomaterials have broad application prospects, their long-term effects with regard to the environment and potential ecological risks are still unclear, and they may have unknown impacts on soil microorganisms and ecosystems. Zero-valent iron treatment may change the soil’s redox potential, adversely affecting some microorganisms and chemical reactions in the soil that rely on specific redox conditions. The effectiveness of soil amendments may vary depending on factors such as soil type, pollution level, and types of ARGs, and the excessive use of certain soil amendments may lead to soil nutrient imbalances or other soil quality issues.

### 4.3. Bioremediation Methods

Bioremediation is a prevalent soil remediation strategy for the abatement of ARGs. Bioremediation involves chemical reactions facilitated by (micro)organisms that degrade or transform pollutants into less toxic or non-toxic forms, thereby repairing or eliminating environmental pollution [126]. Microbial remediation, on the other hand, involves the use of metabolic activities of microorganisms (e.g., bacteria, fungi, actinomycetes, etc.) to degrade or transform organic pollutants in the environment [127]. A study demonstrated that microbial remediation treatments based on the tetracycline-resistant strain *Proteus terrae* ZQ02 resulted in a significant reduction in the number of high-risk ARGs in agricultural soils and contributed to a reduction in the risk of transferring ARGs from soil to corn [128]. Phytoremediation, a common tool in bioremediation, utilizes plants and their inter-root microorganisms to uptake, transform, degrade, or stabilize contaminants in soil [129]. Ginger roots secrete humus-like compounds that reduce heavy metal bioavailability and inhibit ARG transfer via MGEs [108,110]. However, this approach requires careful crop selection, as many plants (e.g., wheat, *Brassica chinensis* L.) inadvertently promote ARGs by altering rhizosphere microbial dynamics [100,130]. In addition, the utilization of certain animal behaviors to enhance soil structure and increase aeration to facilitate the degradation of pollutants in the soil is prevalent in bioremediation [131]. Earthworms are a prevalent organism employed in the context of soil remediation, and a decline in the number and relative abundance of ARGs has been observed in soil ecosystems following the introduction of earthworms [132].

Although biological remediation methods have many advantages in the remediation of soil ARGs, they also face certain limitations. Microbial remediation relies on the growth and metabolic conditions of specific microorganisms, such as temperature, pH value, and nutrients. Minor changes in environmental conditions can significantly affect microbial activity and remediation effectiveness. Moreover, microorganisms may not be able to effectively degrade some complex-structured or high-concentration ARGs. The cycle of phytoremediation is usually long, making it difficult to quickly achieve remediation goals for ARG pollution, which needs to be addressed urgently. Meanwhile, plant growth is also restricted by various factors, including soil conditions and climate. Some plants may also be affected by the toxicity of pollutants, leading to poor growth. When using animal behavior for remediation, the introduction of animals may disturb the existing soil ecosystem. For example, earthworms may alter the physical structure of the soil, affecting the living environment of other soil organisms [132]. Moreover, the reducing effect of animals on ARGs is relatively limited and cannot completely solve severe pollution problems [131].

### 4.4. Integrated Remediation Approaches

In addition to employing the remediation technologies individually to mitigate ARGs in soil, there is a possibility to utilize two or more remediation strategies to enhance pollutant removal efficiency or to address complex environmental problems. This strategy has the capacity to leverage the strengths of diverse technologies while circumventing the constraints imposed by a standalone approach. The effectiveness of different remediation methods in reducing ARGs is summarized in Table 1, which highlights the potential of combined strategies to achieve higher removal rates.

As evidenced by Zheng et al. (2021), the concurrent implementation of pyrophosphoric acid (PA) and biochar, either individually or in a coordinated manner, has been demonstrated to be efficacious in remediating soil contamination in agricultural settings. Furthermore, they suggested that the immobilization of PA or other bacteriostatic agents on biochar by modification methods such as cross-linking of chitosan and slow release of organic anion complexes could increase the efficiency of their co-application in controlling the efficiency of soil ARG contamination [138]. In a related piece of research, Zhang et al. (2022) showed that the joint effect of bacteria and biochar resulted in a decrease in resistance genes and an elevation in the abundance of the genus *Bacillus*. This phenomenon played a part in facilitating the reduction in genes [139]. Duan et al. (2023) discovered that *Bacillus cereus* and nano-biochar enhanced soil properties. They also influenced the abundance of ARGs through directly or indirectly modifying the bacterial community composition in both soil and lettuce. As a result, the transfer of ARGs to the above-ground parts of plants was impeded [140]. In general, there are various strategies to reduce ARGs in agricultural soils, and there are also technologies being researched and developed. Each remediation method has certain advantages and limitations, and their efficacy is inherently constrained by contaminant types (e.g., organic vs. inorganic pollutants) and site-specific soil characteristics such as pH, texture, and organic matter content [141,142]. Therefore, practical implementation requires a holistic evaluation of both pollutant profiles and soil properties to design context-adapted remediation strategies.

Although the integrated remediation method combines the advantages of various technologies, some limitations have also been exposed in practical applications. While the integrated remediation method is theoretically capable of integrating the strengths of multiple technologies, in practice, there may be mutual interference between different remediation methods. For example, certain chemical remediation agents may inhibit the activity of microorganisms in biological remediation, resulting in less-than-ideal remediation outcomes. Moreover, the design and implementation of integrated remediation methods are more complex. They require an in-depth understanding of various remediation technologies, and the costs may also be higher, including material costs, equipment costs, and training costs for technical personnel. These factors, to some extent, restrict its large-scale promotion and application.

## 5. Suggestions and Prospect

The presence of ARGs in agricultural soils poses serious environmental and public health threats. Horizontal gene transfer among microbes can increase resistant strains, reducing antibiotic therapy efficacy. This disrupts soil microbial community balance, affecting soil ecosystem functionality and stability. Crops may absorb antibiotics and ARGs from soil, transmitting them through the food chain and increasing health risks. ARGs also impact soil fertility and agricultural product safety. As resistance rises, treating infectious diseases becomes harder and costlier, with significant socio-economic impacts. Effective management strategies are crucial.

Firstly, future research should focus on the long-term ecological impacts of ARGs in agricultural soils. Studies should investigate how ARGs affect soil microbial community dynamics over the long term, particularly regarding soil health and ecosystem services. Longitudinal studies can help identify the cumulative effects of ARGs on soil biodiversity, nutrient cycling, and overall soil functionality. Additionally, research should explore the potential for ARGs to evolve and adapt in response to environmental pressures, leading to new resistance mechanisms and multidrug resistance.

Secondly, technical guidance on manure treatment is essential. Manure, rich in organic matter and nutrients, is a common organic fertilizer source but may contain antibiotics and ARGs. Effective manure treatment, especially regarding antibiotic and ARG presence, is crucial. Composting can reduce pathogens and ARGs, but its efficacy depends on factors like raw material proportion, moisture regulation, turning frequency, and maturation assessment [143,144]. Providing technical guidance and training to operators and technicians is imperative for safe and effective manure treatment technology implementation. Regular monitoring and evaluation of treatment efficacy, particularly ARG reduction, is also necessary.

Thirdly, developing standards for ARG control in organic fertilizers is vital. Organic fertilizers are a significant ARG source in agricultural soils, and establishing safety thresholds for ARGs in them is crucial for risk mitigation. These thresholds should consider factors like usage frequency, application rates, crop types, and soil characteristics. The monitoring and assessment of ARGs during the production and application of organic fertilizers are of critical importance. In the same vein, regularly monitoring the contamination levels of antibiotics and ARGs in agricultural soils is indispensable. Educating and training farmers, producers, and workers to raise ARG risk awareness and teach best practices is also important.

Lastly, the development and application of soil remediation technologies and cost-effective remediation strategies are vital for reducing ARGs in soil. Bio in situ remediation technologies like phage therapy and biochar application can be researched and applied. Phages target and lyse ARG-bearing bacteria, reducing ARG spread, while biochar adsorbs antibiotic molecules, reducing their bioavailability and ARG selection pressure [145,146]. Research should focus on the optimization of existing technologies and the exploration of novel approaches. For instance, combining biochar with other amendments (e.g., zero-valent iron nanoparticles) could enhance the removal of ARGs and antibiotics. Additionally, the use of engineered microorganisms or synthetic biology approaches could provide targeted solutions for ARG degradation. Combining these technologies can effectively manage soil ARGs. Regular monitoring and evaluation of remediation effectiveness, including ARG abundance, soil microbial community structure, and antibiotic residue levels, is essential during technology application.

In conclusion, the proliferation of ARGs in agricultural soils poses multifaceted risks to environmental stability, public health, and socio-economic systems. To address this crisis, integrated strategies are essential: reducing prophylactic antibiotic use in livestock and agriculture to minimize selection pressure on ARGs; standardizing manure treatment to eliminate residual antibiotics; establishing stringent thresholds for ARGs in organic fertilizers; and advancing remediation technologies such as biochar and electrokinetic methods. Additionally, long-term ecological monitoring and farmer education are critical to curbing ARG dissemination. By prioritizing source control through prudent antibiotic stewardship and implementing multi-layered mitigation measures, sustainable soil management can be achieved, safeguarding ecosystem integrity and human health.

## Figures and Tables

**Table 1:** Comparison of the removal rate of antibiotic resistance genes by different repair methods.

Removal Methods	Removal Rate	Reference
Electrokinetic treatment (0.8 V cm^−1^, polarity reversal interval of 12 h)	54.6%	[133]
hyperthermophilic (60–95 °C)	98.8%	[134]
Mesophilic static composting (50 °C)	57%	[135]
Electrochemically driven UV/Cl_2_ process	99.9%	[136]
Biochar (BPL14 and NPL14)	88.6%	[124]
Combination of bacterial and sophorolipid	98.1%	[137]

## Data Availability

No new data were created or analyzed in this study. Data sharing is not applicable to this article.
